# Synthesis and antibacterial activity of new 1,2,3-triazolylmethyl-2*H*-1,4-benzothiazin-3(4*H*)-one derivatives

**DOI:** 10.1186/s13065-018-0494-2

**Published:** 2018-11-29

**Authors:** Mohamed Ellouz, Nada Kheira Sebbar, Ismail Fichtali, Younes Ouzidan, Zakaria Mennane, Reda Charof, Joel T. Mague, Martine Urrutigoïty, El Mokhtar Essassi

**Affiliations:** 1 Laboratoire de Chimie Organique Hétérocyclique, Centre de Recherche des Sciences des Médicaments, Pôle de Compétences Pharmacochimie, Faculté des Sciences, Mohammed V University in Rabat, Av. Ibn Battouta, BP 1014, Rabat, Morocco; 2 Laboratoire de Chimie Organique Appliquée, Faculté des Sciences et Techniques, Université Sidi Mohamed Ben Abdallah, Route Immouzer, Fès, Morocco; 3 Département de bactériologie, Institut national d’hygiène, Avenue Ibn Batouta, Agdal, B.P. 769, 11000 Rabat, Morocco; 4 Department of Chemistry, Tulane University, New Orleans, LA 70118 USA; 5 CNRS, LCC (Laboratoire de Chimie de Coordination), 205, Route de Narbonne, 31077 Toulouse, France; 6 UPS, INPT, LCC, Université de Toulouse, 31077 Toulouse, France; 7 Laboratoire de Chimie Bioorganique Appliquée, Faculté des Sciences, Université Ibn Zohr, Agadir, Morocco; 8 Moroccan Foundation for Advanced Science, Innovation and Research (MASCIR), Rabat, Morocco

**Keywords:** 1,2,3-Triazole, 1,4-Benzothiazine, Antimicrobial activity, Cycloaddition, Spectroscopic methods

## Abstract

**Background:**

A novel series of 1,2,3-triazole derivatives containing 1,4-benzothiazin-3-one ring (**7a**–**9a**, **7b**–**9b)**, (**10a**–**12a**, **10b**–**12b)** and (**13**–**15**) were synthesized by 1,3-dipolar cycloaddition reactions of azides α-d-galactopyranoside azide **F**, 2,3,4,6-tetra-*O*-acetyl-(d)-glucopyranosyl azide **G** and methyl-*N*-benzoyl-α-azidoglycinate **H** with compounds **4**–**6**.

**Findings:**

Initially, the reactions were conducted under thermal conditions in ethanol. The reaction leads, each time, to the formation of two regioisomers: (Schemes 2, 3) with yields of 17 to 21% for 1,5-disubstituted 1,2,3-triazole-regioisomers (**7b**–**12b**) and yields ranging from 61 to 65% for the 1,4-disubstituted regioisomers (**7a**–**12a**). In order to report an unequivocal synthesis of the 1,4-regioisomers and confirm the structures of the two regioisomers obtained in thermal conditions (Huisgen reactions), the method click chemistry (Copper-Catalyzed Azide-Alkyne Cycloaddition) has been used.

**Conclusions:**

The newly synthesized compounds using cycloaddition reactions were evaluated in vitro for their antibacterial activities against some Gram positive and Gram negative microbial strains. Among the compounds tested, the compound **8a** showed excellent antibacterial activities against *PA ATCC* and *Acin ESBL* (MIC = 31.2 μg/ml).

## Introduction

**Fig. 1:**
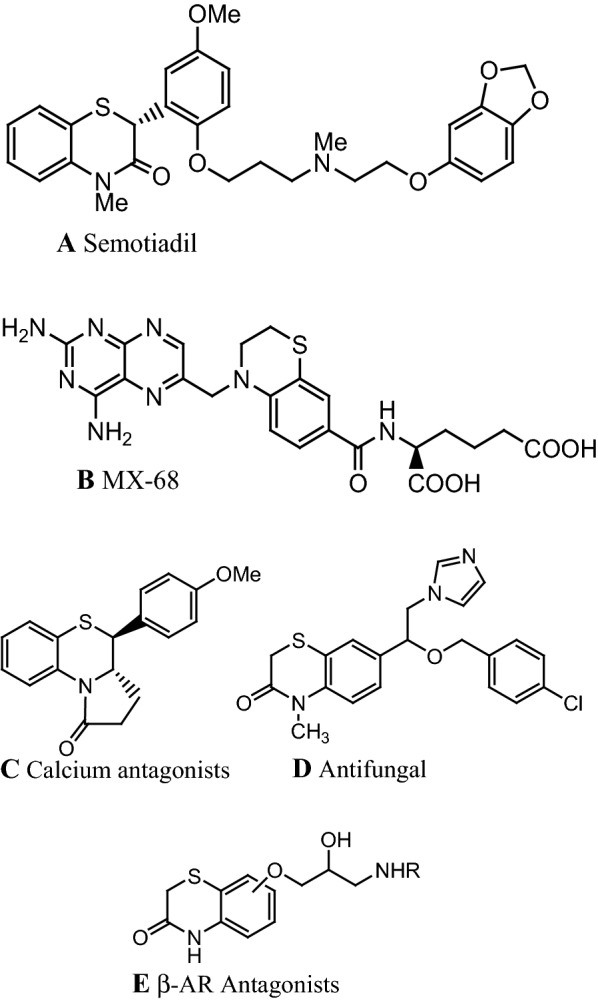
Examples of bioactive molecules derived from 1,4-benzothiazine

Compounds containing 1,4-benzothiazine backbone have been studied extensively both in academic and industrial laboratories. These molecules exhibit a wide range of biological applications indicating that 1,4-benzothiazine moiety is a template potentially useful in medicinal chemistry research and therapeutic applications such as anti-inflammatory [1, 2], antipyretic [3], anti-microbial [4–7], anti-viral [8], herbicide [9], anti-cancer [10–13], and anti-oxidant [14] areas. They have also been reported as precursors for the synthesis of compounds [15] possessing anti-diabetic [16] and anti-corrosion activities [17, 18]. Figure 1 gives some examples of bioactive molecules with 1,4-benzothiazine moieties.

In order to prepare new heterocyclic systems with biological applications, we report in the present work 1,3-dipolar cycloaddition reactions [19–21] between 4-propargyl-2-(substituted)-1,4-benzothiazin-3-ones **4**–**6** as dipolarophiles and α-d-galactopyranoside azide **F** or 2,3,4,6-tetra-*O*-acetyl-(d)-glucopyranosyl azide **G** or methyl-*N*-benzoyl-α-azidoglycinate **H** as dipoles. It is worthy to note that the integration of two or more active heterocyclic rings in the same molecule may lead to new hybrid with broad biological activities.

As a continuation of our previous works related to the synthesis of new heterocyclic systems with potent pharmacological properties we describe a novel 1,2,3-triazol-α-d-galactopyranoside-2-(substituted)-1,4-benzothiazin-3-one (**7a**–**9a**, **7b**–**9b)**, 1,2,3-triazol-2,3,4,6-tetra-*O*-acetyl-(d)-glucopyranosyle-2-(substituted)-1,4-benzothiazin-3-one (**10a**–**12a**, **10b**–**12b)** and 4-[1,2,3-triazolylmethyl]-2-(substituted)-1,4-benzothiazin-3-one (**13**–**15**) derivatives obtained via thermal 1,3-dipolar cycloaddition reactions and click chemistry. [Copper-Catalyzed Azide-Alkyne Cycloaddition (CuAAC)].

## Results and discussion

### Synthesis of dipolarophiles 4–6

**Scheme 1:**
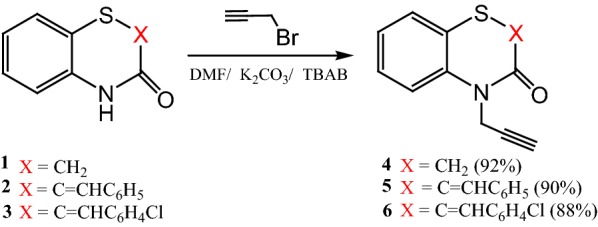
Synthesis of dipolarophiles **4**–**6**

Dipolarophiles **4**–**6** have been prepared with good yields (88–92%) via alkylation réactions of compounds **1**–**3** by propargyl bromide under phase transfer catalysis conditions using tetra-*n*-butylammonium bromide (TBAB) as catalyst and potassium carbonate as base in dimethylformamide at room temperature (Scheme 1).

**Fig. 2:**
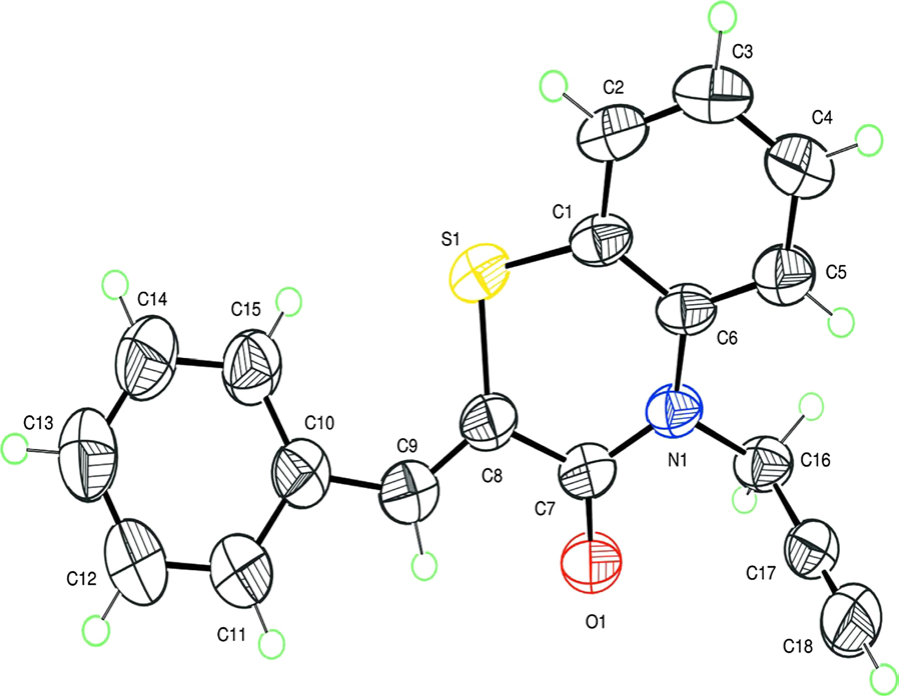
The structure of compound 5, showing the atom-umbering scheme, with displacement ellipsoids drawn at the 30% probability level

The structures of compounds isolated have been identified on the basis of ^1^H NMR and ^13^C NMR spectral data. The ^1^H NMR spectrum of the compounds **4**–**6** in DMSO d_6_ shows signals for the propargyl group as a doublet at 4.74, 4.90 and 4.86 ppm, respectively and a triplet centered at 2.20 (2.21) and 3.31 ppm corresponding to methylene groups bonded to the nitrogen atom and acetylenic HC≡C–proton, respectively. The ^13^C NMR spectrum showed the signal of hydrogenated acetylenic carbon at 75.0, 75.5 and 75.47 ppm, respectively. The structures of compounds **4** and **5** were confirmed by a crystallographic studies [22, 23] (Fig. 2).

The crystallographic study confirms that compounds **5**, **6** have Z configuration about the exocyclic double bond. This result will allow to assign the Z configuration to all compounds coming from the products **5**, **6** in future ulterior cycloaddition reactions the dipolarophiles **4**–**6** are then involved in cycloaddition reactions with the dipoles given above leading to new benzothiazine derivatives containing various 1,2,3-triazole moieties able to modulate their biological activities [24, 25].

### Synthesis of new 1, 2, 3-triazolylmethyl-2H-1,4-benzothiazin-3(4*H*)-one derivatives

**Scheme 2:**
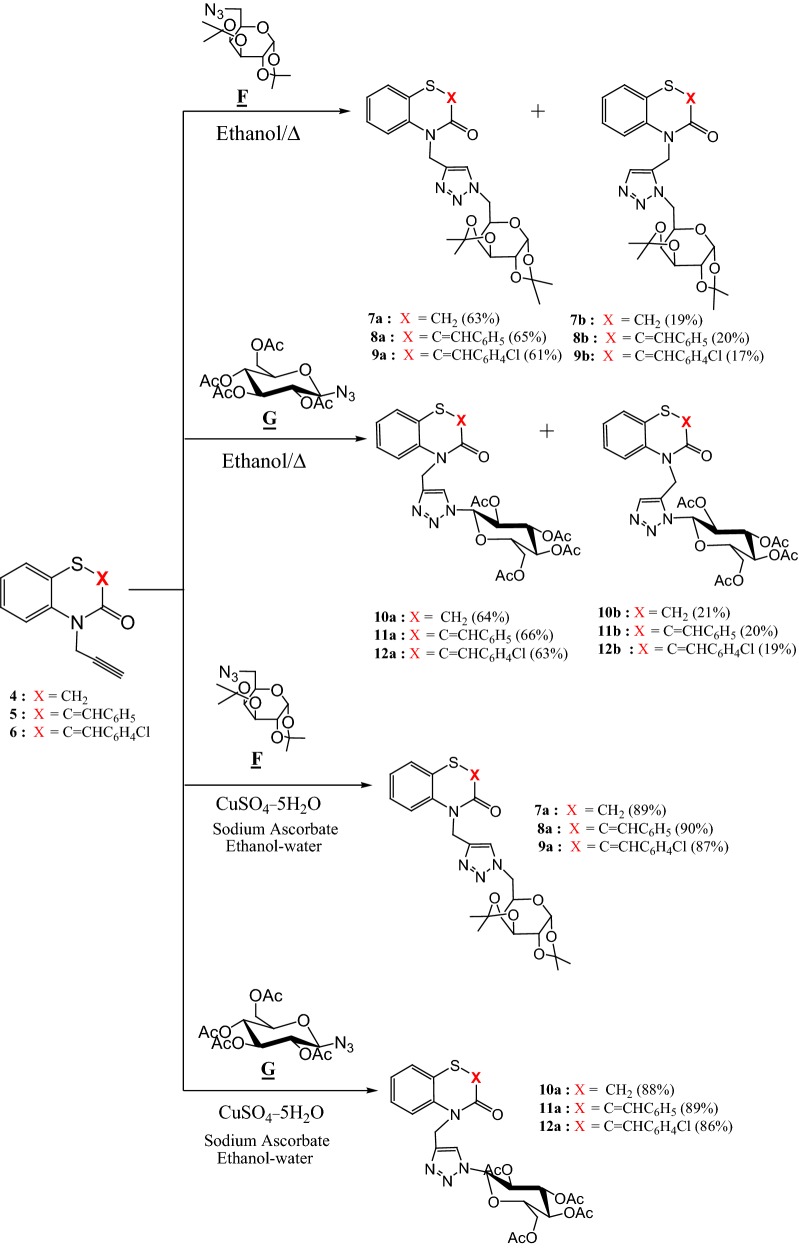
Preparation of new 1,2,3-triazolylmethyl-2*H*-1,4-benzothiazin-3-one derivatives

The literature reports several studies concerning the synthesis of 1,4 or 1,5-disubstituted 1,2,3-triazoles according to the Huisgen method under thermal conditions [26]. Due to the importance of the 1,2,3-triazole moiety in the biological and therapeutic areas, it seems interesting to include this backbone in the 1,4-benzothiazine derivatives. Thus, we have studied the reaction between azides F, G and H and compounds **4**–**6**. The reaction was conducted in hot ethanol leading to the formation of products **7**–**12** related in each case to two regioisomers (**7a**–**12a** and **7b**–**12b**) using azides **F**, **G**. The yields are between 17 and 21% for 1,5-disubstituted 1,2,3-triazole-regioisomers (**7b**–**12b**) and between 61 and 65% for 1,4-disubstituted regioisomers (**7a**–**12a**). These results are in agreement with those described in the literature [27–30]. The two 1,4 and 1,5 disubstituted 1,2,3-triazole isomers have been separated by chromatography on silica gel column [eluent: ethyl acetate/hexane (1/9)] (Scheme 2).

In order to report an unequivocal synthesis of the 1,4-regioisomers **7a**–**12a** and confirm the structures of the two regioisomers obtained previously in thermal conditions (Huisgen reactions), the method click chemistry [Copper-Catalyzed Azide-Alkyne Cycloaddition (CuAAC)] described in the literature [31–34] has been used in the condensation of dipolarophiles **4**–**6** with azides **F** and **G** in the presence of copper (II) sulfate (CuSO_4_), sodium ascorbate as a reducing agent in water and ethanol mixture (1:1). Thus the 1,4-disubstituted 1,2,3-triazole derivatives **7a**–**12a** have been obtained exclusively in 86 to 90% yields. All the products are fully characterized by ^1^H and ^13^C NMR (see “Experimental part”). ^1^H NMR spectra in DMSO d_6_ of compounds **7a**–**12a** present in particular signals: as singlets at 4.33(**7a**), 4.49(**8a**), 4.55(**9a**), 4.37(**10a**), 4.34(**11a**) and 4.37(**12a**) ppm related to the two protons of the methylene group linked to the nitrogen atom of 1,4-benzothiazine moiety and a signals as singlets at 7.93(**7a**), 8.01(**8a**), 7.99(**9a**), 8.35(**10a**), 8.37(**11a**) and 8.39(**12a**) ppm corresponding to the proton in position 5 of the 1,2,3-triazole ring. The ^1^H NMR spectra of 1,5-disubstituted regioisomers **7b**–**12b** exhibit particularly signals as a singlets at 4.54(**7b**), 4.39(**8b**), 4.42(**9b**), 4.37(**10b**), 4.34(**11b**) and 4.34(**12b**) ppm due to the two protons of the methylene groups linked to the nitrogen atom in position 1 of the 1,4-benzothiazine ring and signals as singlets at 8.31(**7b**), 8.29(**8b**), 8.25(**9b**), 7.63(**10b**), 7.62(**11b**) and 7.61(**12b**) ppm related to the proton in position 4 of the 1,2,3-triazole moiety. The ^13^C NMR spectra of compounds **7a**–**12a** highlight in particular the signals of the two methylene groups linked to the nitrogen atom in position 3 of the bicyclic system at 40.78(**7a**), 41.57(**8a**), 41.42(**9a**), 41.84(**10a**), 41.51(**11a**) and 40.99 (**12a**) ppm, and for compounds **7b**–**12b** the signals at 41.00(**7b**), 39.77(**8b**), 39.23(**9b**), 41.84(**10b**), 41.84(**11b**) and 41.74(**12b**) ppm. These results are in good agreement with those observed in the literature which show that the proton signal at position 5 of the 1,2,3-triazole ring is more deshielded than the one for the proton at position 4 of 1,2,3-triazole for compounds **7b**–**12b** [27–30].

**Scheme 3:**
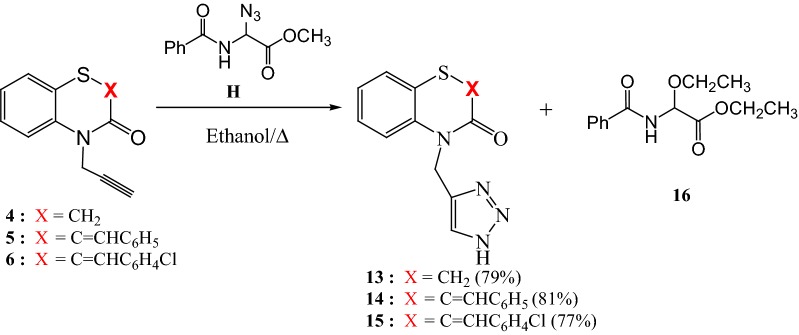
Preparation of new 1,2,3-triazoles monosubstituted **13**–**15**

It should be noted that when compounds **4**–**6** reacted with azide **H** it has allowed us to isolate in each case only one isomer **13**–**15** (Scheme 3) with yields between **77** and 83%. For compounds **13**–**15** the ^1^H NMR in DMSO d_6_ exhibit in particular signals as singlets at 5.16(**13**), 4.86(**14**) and 4.85(**15**) ppm related to the two protons of methylene group linked to the nitrogen atom at position 4 and a singlets at 7.40(**13**), 7.54(**14**) and 7.53(**15**) ppm corresponding to the proton in position 5 of the 1,2,3-triazole moiety. The ^13^C NMR spectra highlight in particular the presence of signals related to the methylene groups at 40.32(**13**), 35.47(**14**) and 35.01(**15**) ppm.

**Fig. 3:**
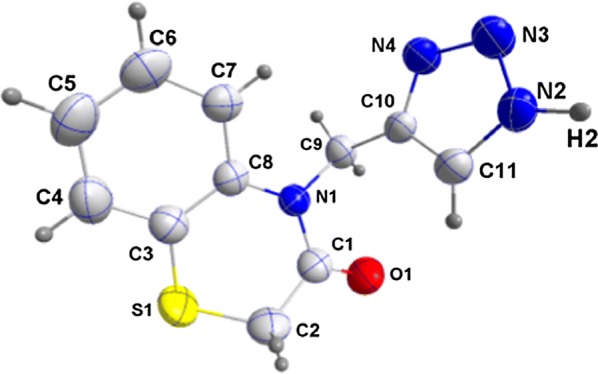
Molecular structure of the compound 13 with the atom-labelling scheme. Displacement ellipsoids are drawn at the 50% probability ellipsoids (CCDC 1564624)

**Fig. 4:**
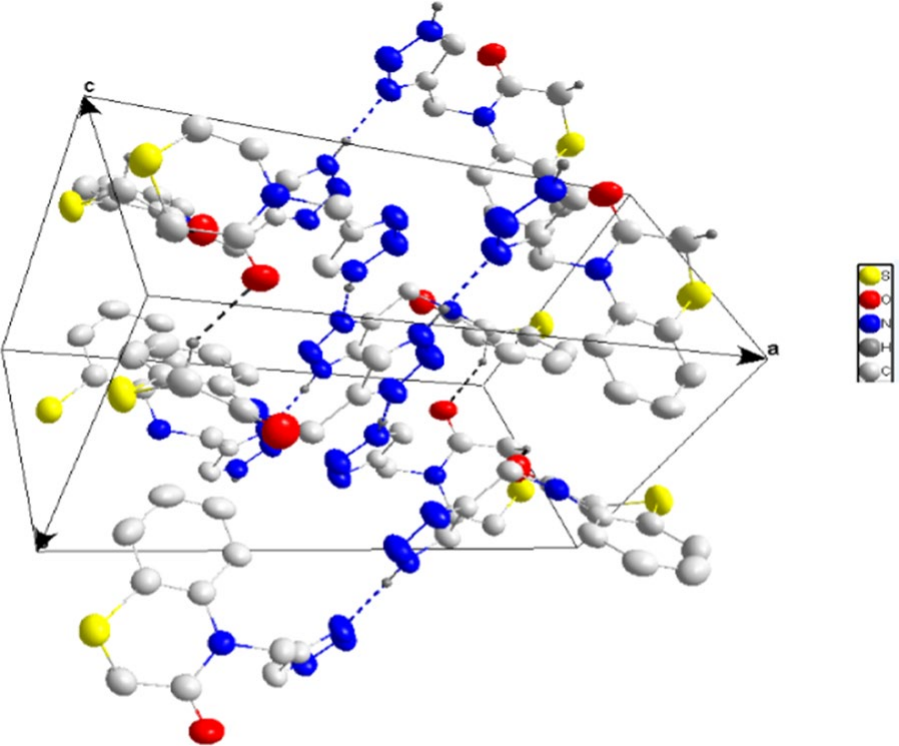
Packing showing portions of the chains formed by N–H···N hydrogen bonds (blue dotted lines) and their association through C–H···O hydrogen bonds (black dotted lines) of compound **13**

The crystallographic analysis of compound **13** indicates that the triazole nitrogen atom is unsubstituted and confirms the structures of compounds **13**–**15** (Figs. 3 and 4). It is interesting to note that compound **13** crystallizes in monoclinic system (P2_1_/c). The crystallographic data have been assigned to the deposition number. CCDC 1564624.

**Scheme 4:**
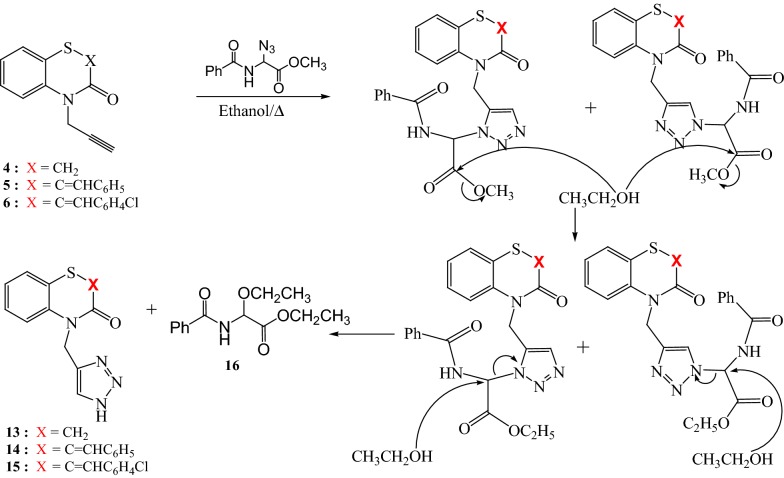
Proposed mechanism for the formation of 1H-4-substituted 1,2,3-triazoles **13**–**15**

The formation of compounds **13**–**15** suggests that the reaction operates via a traditional mechanism of 1,3-dipolar cycloaddition of azide **H** with alkynes **4**–**6**, followed by a transesterification. The nucleophilic substitution of triazole unit by ethanol leads to compounds **13**–**15** next to the glycine derivative **16**, Scheme 4.

## Biological evaluation in vitro antibacterial evaluation

**Fig. 5:**
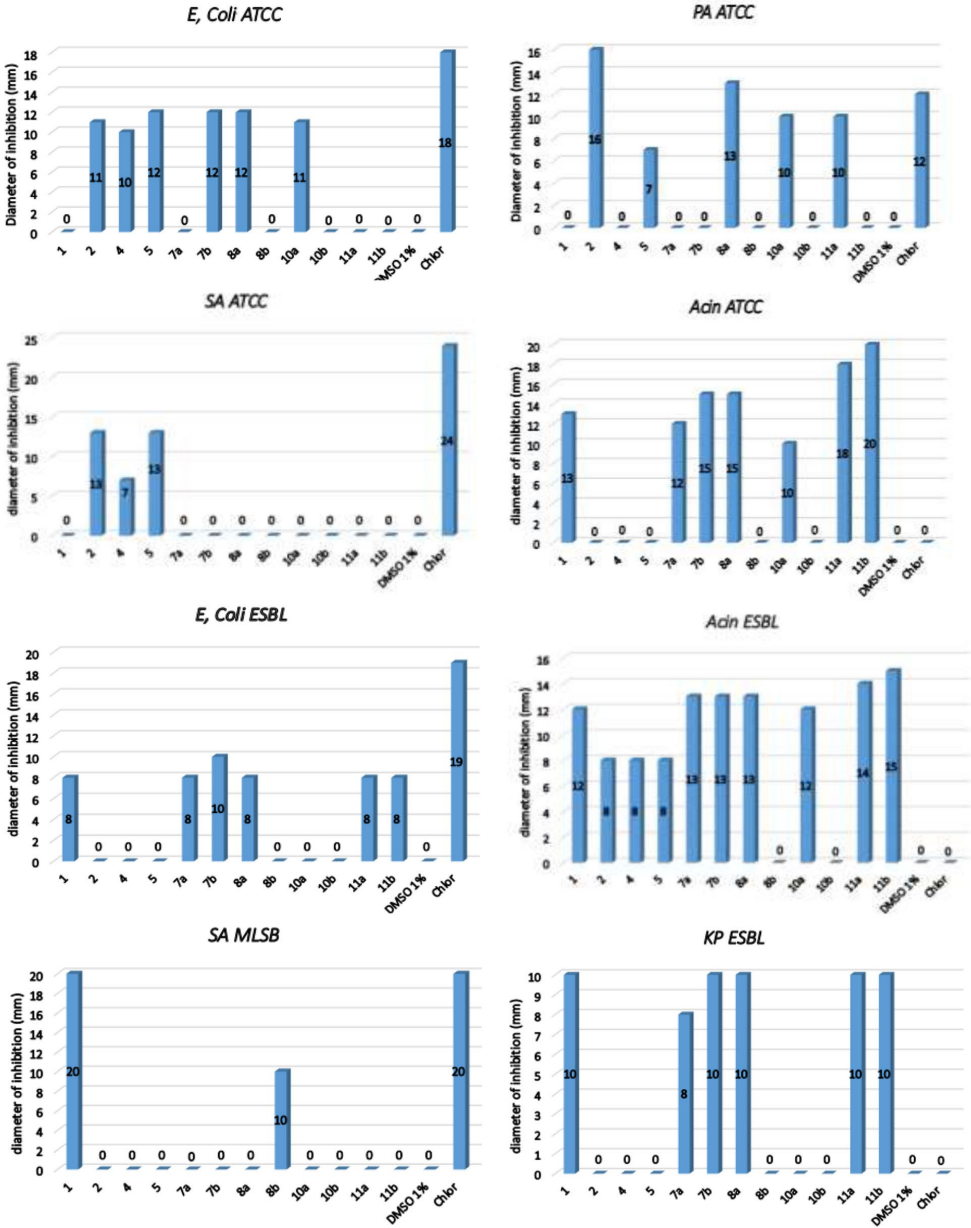
Results of the antibacterial activity of the synthesized compounds **1**, **2**, **4**, **5**, **7a**, **7b**, **8a**, **8b**, **10a**, **10b**, **11a** and **11b** vis-a-vis bacteria tested (*Escherichia coli* ATCC, *Pseudomonas aeruginosa* ATCC, *Staphylococcus aureus* ATCC, Acinetobacter ATCC, *Escherichia coli BLSE,* Acinetobacter BLSE, *Staphylococcus aureus* MLSB and *Klebsiella pneumonia BLSE*). *Chlor* chloramphenicol (30 µg/ml), *DMSO* dimethylsulfoxide (1%)

The compounds tested showed an average antibacterial activity and the results of the assessments are shown in Fig. 5 and Table 1.

**Table 1:** Results of the in vitro antibacterial activity (MIC values µg/ml) of the synthesized compounds **1**, **2**, **4**, **5**, **7a**, **7b**, **8a**, **8b**, **10a**, **10b**, **11a** and **11b** vis-a-vis bacteria tested (*Escherichia coli* ATCC, *Pseudomonas aeruginosa* ATCC, *Staphylococcus aureus* ATCC, Acinetobacter ATCC, *Escherichia coli BLSE,* Acinetobacter BLSE, *Staphylococcus aureus* MLSB and *Klebsiella pneumonia BLSE*)

	*E. coli* ATCC	PA ATCC	SA ATCC	Acin ATCC	*E. coli* ESBL	Acin ESBL	SA MLSB	KP ESBL
**1**	–	–	–	62.5	250	31.25	250	250
**2**	250	125	125	–	–	250	–	–
**4**	125	–	250	–	–	250	–	–
**5**	125	250	250	–	–	250	–	–
**7a**	–	–	–	250	125	62.5	–	62.5
**7b**	125	–	–	125	62.5	62.5	–	62.5
**8a**	62.5	62.5	–	125	125	62.5	–	125
**8b**	–	–	–	–	–	–	125	–
**10a**	125	125	–	125	–	62.5	–	–
**10b**	–	–	–	–	–	–	–	–
**11a**	–	125	–	125	62.5	125	–	62.5
**11b**	–	–	–	250	62.5	125	–	62.5
DMSO	–	–	–	–	–	–	–	–
Chlor	4	7.5	2.5	–	5	–	3	–

*Chlor* chloramphenicol (30 µg/ml), *DMSO* dimethylsulfoxide (1%)

The results are presented in the form of antibiograms below:

The newly synthesized compounds **7a**(**7b**), **8a**(**8b**), **10a**(**10b**) and **11a**(**11b**), have been tested for their antibacterial activity in vitro against two Gram-positive bacteria: *Staphylococcus aureus ATCC 25923* and *Staphylococcus aureus MLSB* and six Gram-negative bacteria: *Escherichia coli* (*E. coli*) *ATCC 25922*, *Pseudomonas aeruginosa* (*PA*) *ATCC 27853*, *Acinetobacter* (*Acin*) *ATCC 17978*, *Escherichia coli ESBL*, *Klebsiella pneumonia* (*KP*) *ESBL* and *Acinetobacter ESBL*. The compounds were tested at a concentration of 500 µg/ml, using disc diffusion method [35], the minimum inhibitory concentration (MIC) was measured in µg/ml and compared with that of chloramphenicol as reference standard. The strains used in this work are widely encountered in various pathologies in humans, were obtained from the Department of Microbiology, National Institute of Hygiene, Rabat, Morocco.

The results obtained in the antibacterial activity of the compounds **1**–**2**, **4**–**5**, **7a(7b), 8a(8b), 10a(10b)** and **11a(11b)** showed better activity vis-a-vis the eight tested bacteria (Table 1). This study determined the MIC of some synthesized derivatives of 1,4-benzothiazine. The results of the antibacterial activity of the products tested showed the absence of growth inhibition for compound **1** in the three bacterial strains: *Escherichia coli* (ATCC), *Pseudomonas aeruginosa* (ATCC) and *Staphylococcus aureus* (ATCC) and an activity MIC = 31.25 µg/ml for *Acinetobacter* (BLSE), MIC = 62.5 µg/ml for *Acinetobacter* (ATCC) and MIC = 250 µg/ml for *Escherichia coli* (BLSE), *Staphylococcus aureus* (MLSB) and *Klebsiella pneumonia* (BLSE). By against the compound **2** obtained by substituting the compound **1** by the benzylidene group in position 2 has caused an activity MIC = 125 μg/ml for *Pseudomonas aeruginosa* (ATCC), *Staphylococcus aureus* (ATCC) and a MIC = 250 μg/ml *Escherichia coli* (ATCC) and *Acinetobacter* (BLSE) with absence of growth inhibition for compound **2** in four bacterial strains *Acinetobacter* (ATCC), *Escherichia coli* (ESBL), *Staphylococcus aureus* (MLSB) and *Klebsiella pneumoniae* (BLSE). In order to increase the inhibitory activity of compounds **1** and **2** we alkylated those compounds with propargyl bromide. It is deducible that the presence of a prop-1-yn group in compounds **4** and **5** provides a better growth inhibition activity for compound **4** against three bacterial strains tested with MIC of 125 μg/ml for *Escherichia coli* (ATCC), MIC = 250 μg/ml for *Staphylococcus aureus* (ATCC), *Acinetobacter* (ESBL), with lack of growth inhibition in the two bacterial strains tested *Pseudomonas aeruginosa* (ATCC), *Acinetobacter* (ATCC), *Escherichia coli* (ESBL), *Staphylococcus aureus* (MLSB) and *Klebsiella pneumoniae* (BLSE). On the other hand the compound **5** has no activity against four bacterial strains tested: *Acinetobacter* (ATCC), *Escherichia coli* (ESBL), *Staphylococcus aureus* (MLSB) and *Klebsiella pneumoniae* (BLSE). However, the compound **5** also presents an activity with MIC of the order of 125 μg/ml for *Escherichia coli* (ATCC) and 250 μg/ml for *Pseudomonas aeruginosa* (ATCC), *Staphylococcus aureus* (ATCC) and *Acinetobacter* (BLSE).

Also, for the eight products triazole **7a(7b), 8a(8b), 10a(10b)** and **11a(11b)** obtained by cycloaddition reactions, it is worthy to note that compound **8a** obtained by cycloaddition with azide **F** possess a strong inhibitory activity during the treatment of different bacteria: CMI = 62.5 µg/ml for *Escherichia coli* (ESBL), *Pseudomonas aeruginosa* (ATCC), *Acinetobacter* (ESBL) and CMI = 125 µg/ml for *Acinetobacter (*ATCC), *Escherichia coli* (ESBL), *Klebsiella pneumoniae* (ESBL).

Finally the compound **10b** obtained by cycloaddition with azide **G** the results of the antibacterial activity of the products tested showed the absence of growth inhibition for compound **10b** towards all tested bacteria. In general, the molecular specifications of the 1,2,3-triazoles can also be used as a linker and show bioisosteric effects on peptide linkage, aromatic ring, double bonds. Some unique features like hydrogen bond formation, dipole–dipole and π stacking interactions of triazole compounds have increased their importance in the field of medicinal chemistry as they bind with the biological target with high affinity due to their improved solubility. This study is expected to take anti-inflammatory tests, antifungal, antiparasitic and anti-cancer, because the literature gives a lot of interesting results on these topics. Also, other bacteria should be selected to expand the investigation [36–38]. The 1,2,3-triazole based heterocycles have been well exploited for the generation of many medicinal scaffolds exhibiting anti-HIV, anticancer, antibacterial activities.

## Conclusion

In conclusion, in the development of this work, the synthesis of the new heterocyclic systems derived from 1,2,3-triazolyl-1,4-benzothiazin-3-one was carried out in satisfactory yields by cycloaddition reactions under thermal and catalytic conditions (Cu I). The results showed a periselectivity and regioselectivity as a function of the dipole (azides **F**, **G** and **H**) employed. In addition, the obtained results highlight an original synthesis reaction of 1,2,3-triazoles monosubstituted by the action of azide-glycine (**H**) on dipolarophiles **4**–**6** under thermal conditions. The heterocyclic systems obtained were identified by ^1^H NMR, ^13^C NMR, and confirmed for product **13** by X-ray diffraction. The synthesized products were subjected to the evaluation of antibacterial activity. Several compounds tested showed significant activity.

## Experimental part

General: Column chromatography was performed on silica gel 60 (Merck 230–400 mesh). Nuclear magnetic resonance spectra were recorded on a Varian Unity Plus spectrometer ^1^H NMR at 300 MHz; the chemical shifts (d) are expressed in parts per million (ppm) and the coupling constants (J) in Hertz (Hz). DMSO was used as the solvent and SiMe4 as the reference.

### General procedure of synthesis compounds 4, 5 and 6

To a solution of (6.05 mmol) of 2-substituted)-1,4-benzothiazin-3-one **1** (**2 or 3**) in 15 ml of DMF, were added 11.3 mmol of potassium carbonate. The reaction mixture was stirred magnetically for 5 min then added 0.6 mmol of bromide tetra-*n*butylammonium (BTBA) and 7.26 mmol of propargyl bromide, then the mixture was stirred magnetically for 24 h. After removal of salts by filtration, the solution was evaporated under reduced pressure, and the residue obtained is dissolved in dichloromethane. The remaining salts are extracted with distilled water, and the mixture obtained was chromatographed on silica gel column [eluent: ethyl acetate/hexane (1/9)].

#### 4-(Prop-2-ynyl)-3,4-dihydro-2H-1,4-benzothiazin-3-one **4**

Yield: 92%; mp = 492 K; ^1^H-NMR (DMSO-_d6_, 300 MHz) δ [ppm]: 7.42–7.04 (m, 4H, H_arom_), 4.74 (d, 2H, J = 1.9 Hz NCH_2_), 3.55 (s, 2H, S-CH_2_), 2.20 (t, 1H, J = 1.9 Hz ≡ CH,); ^13^C-NMR (DMSO-_d6_, 62.5 MHz) δ [ppm]: 165.2 (C=O), 139.0, 123.4, 79.8 (Cq), 128.6, 128.0, 124.1, 118.5 (CH_arom_), 75.0 (≡CH), 33.8 (NCH_2_), 30.6 (S-CH_2_).

#### (2Z)-2-Benzylidene-4-(prop-2-ynyl)-3,4-dihydro-2H-1,4-benzothiazin-3-one **5**

Yield: 90%; mp = 403 K; ^1^H-NMR (DMSO-_d6_, 300 MHz) δ [pm]: 7.84 (s, 1H, CHvinyl), 7.66–7.09 (m, 9H, H_arom_), 4.90 (d, 2H, J = 1.8 Hz, NCH_2_), 2.21 (t, 1H, J = 1.8 Hz, ≡CH). ^13^C-NMR (DMSO-_d6_, 62.5 MHz) δ [ppm]: 161.0 (C=O), 135.8, 134.4, 134.3, 118.4, 79.6 (Cq), 135.5 (CH_vinyl_), 130.6, 129.8, 129.1, 128.1, 126.8, 124.5, 117.8 (CH_arom_), 75.5 (≡CH), 35.0 (NCH_2_).

#### (Z)-2-(4-Chlorobenzylidene)-4-(prop-2-ynyl)-2H-1,4-benzothiazin-3-one **6**

Yield: 88%; mp = 385 K; ^1^H-NMR (DMSO-_d6_, 300 MHz) δ [ppm]: 7.83 (s, 1H, CHvinyl), 7.69–7.11 (m, 8H, H_arom_), 4.86 (d, 2H, J = 1.9 Hz, NCH_2_), 3.31 (t, 1H, J = 1.9 Hz ≡CH). ^13^C-NMR (DMSO-_d6_, 62.5 MHz) δ [ppm]: 161.0 (C=O), 135.77 (CH_vinyl_), 134.08, 134.28, 133.25, 121.04, 118.05 (Cq), 132.3, 129.12, 128.14, 126.86, 124.55, 117.85 (CH_arom_), 75.47 (≡CH), 35.02 (NCH_2_).

### General procedure for the synthesis of compounds 7a–12a, 7b–12b and 13–15 via Huisgen 1,3-dipolar cycloaddition reactions

To a solution of dipolarophile **4 (5** or **6)** (8 mmol) in absolute ethanol (20 ml) was added azide **F** (**G** or **H)** (16 mmol). The reaction mixture was stirred at reflux and the reaction monitored by thin layer chromatography (TLC). After concentration under reduced pressure, the residue was purified by column chromatography on silica gel using a mixture [ethyl acetate/hexane (1/9)] as eluent.

### General procedure for the synthesis of compounds 7a–12a by click chemistry: [Copper-Catalyzed Azide-Alkyne Cycloaddition (CuAAC)]

To a solution of 1 mmol of compound **4 (5** or **6)** and 2 mmol of azide **F** (**G)** in 15 ml of ethanol were added 0.5 mmol of CuSO_4_ and 1 mmol of sodium ascorbate dissolved in 7 ml of distilled water. The reaction mixture was stirred for 24 h at room temperature. The reaction was monitored by TLC. After filtration and concentration of the solution under reduced pressure the residue obtained was chromatographed on silica gel column using as eluent ethyl acetate/hexane (1/9). The compounds have been obtained with yields ranging from **86** to **90**%.

#### 4-[(1′-1″,2″:3″,4″-Di-*O*-isopropylidene-α-d-galactopyranosid-6″-yl)-1′,2′,3′-triazol-4′-yl)methyl]-2*H*-1,4-benzothiazin-3-one **7a**

Yield: 63%; brown oil; ^1^H-NMR (DMSO-_d6_, 300 MHz) δ [ppm]: 1.40, 1.31, 1.30, 1.23 (s, 12H, 4CH_3_), 3.52 (s, 2H, CH_2_–S), 4.69, 4.53, 4.39, 4.22 (m, 4H, 4CH, H_2_, H_3_, H_4_, H_5_), 4.35 (d, 2H, CH_2_–N), 5.32 (d, 2H, CH_2_–N, H_6_), 5.47 (d, 1H, CH, H_1_), 7.55–7.03 (m, 4H, H_arom_), 8.31 (s, 1H, CH_triazole_); ^13^C-NMR (DMSO-_d6_, 62.5 MHz) δ [ppm]: 164.04 (CO), 142.78, 140.17, 123.50, 109.62, 108.29 (Cq), 128.89 (CH_triazole_), 127.39, 124.69, 124.23, 119.00 (CH_arom_), 97.01, 71.74, 70.75, 69.96, 66.97 (5CH, C_1_, C_2_, C_3_, C_4_, C_5_), 50.26, 41.00 (CH_2_–N), 31.23 (CH_2_–S), 26.34, 25.81, 25.27, 24.95 (4CH_3_);

#### 4-[(1′-1″,2″:3″,4″-Di-*O*-isopropylidene-α-d-galactopyranosid-6″-yl)-1′,2′,3′-triazol-5′-yl) methyl]-2*H*-1,4-benzothiazin-3-one **7b**

Yield: 19%; brown oil; ^1^H-NMR (DMSO-_d6_, 300 MHz) δ [ppm]: 1.38, 1.29, 1.28, 1.21 (s, 12H, 4CH_3_), 3.52 (s, 2H, CH_2_–S), 4.62, 4.50, 4.33, 4.15 (m, 4H, 4CH, H_2_, H_3_, H_4_, H_5_), 4.33 (d, 2H, CH_2_–N), 5.12 (d, 2H, CH_2_–N, H_6_), 5.38 (d, 1H, CH, H_1_), 7.50–7.00 (m, 4H, H_arom_), 7.93 (s, 1H, CH_triazole_); ^13^C-NMR (DMSO-_d6_, 62.5 MHz) δ [ppm]: 165.24 (CO), 143.56, 139.84, 123.27, 109.31, 108.60 (Cq), 128.46 (CH_triazole_), 127.76, 124.49, 123.91, 118.63 (CH_arom_), 95.96, 71.04, 70.59, 70.16, 67.26 (5CH, C_1_, C_2_, C_3_, C_4_, C_5_), 50.58, 40.78 (CH_2_–N), 30.79 (CH_2_–S), 26.34, 26.05, 25.27, 24.70 (4CH_3_);

#### (2Z)-2-Benzylidene-4-[(1′-1″,2″:3″,4″-di-*O*-isopropylidene-α-d-galactopyranosid-6″-yl)-1′,2′,3′-triazol-4′-yl)methyl]-2*H*-1,4-benzothiazin-3-one **8a**

Yield: 65%; brown oil; ^1^H-NMR (DMSO-_d6_, 300 MHz) δ [ppm]: 1.41, 1.33, 1.31, 1.25 (s, 12H, 4CH_3_), 4.67, 4.39, 4.38, 4.36 (m, 4H, 4CH, H_2_, H_3_, H_4_, H_5_), 4.39 (d, 2H, CH_2_–N), 5.47 (d, 2H, CH_2_–N, H_6_), 5.32 (d, 1H, CH, H_1_), 7.67–7.06 (m, 4H, H_arom_), 7.85 (s, 1H, CH_vinyl_), 8.29 (s, 1H, CH_triazole_); ^13^C-NMR (DMSO-_d6_, 62.5 MHz) δ [ppm]: 161.44 (CO), 136.06, 134.68, 134.51, 132.47, 130.06,109.44, 108.74 (Cq), 135.26 (CH_vinyl_), 132.47 (CH_triazole_), 130.61, 129.72, 129.08, 127.95, 126.85, 124.49, 118.06 (CH_arom_), 96.12, 70.90, 70.62, 70.22, 68.37 (5CH, C_1_, C_2_, C_3_, C_4_, C_5_), 48.56, 39.77 (CH_2_–N), 26.43, 26.13, 25.27, 24.85 (4CH_3_).

#### (2Z)-2-Benzylidene-4-[(1′-1″,2″:3″,4″-di-*O*-isopropylidene-α-d-galactopyranosid-6″-yl)-1′,2′,3′-triazol-5′-yl)methyl]-2*H*-1,4-benzothiazin-3-one **8b**

Yield: 20%; brown oil; ^1^H-NMR (DMSO-_d6_, 300 MHz) δ [ppm]: 1.37, 1.27, 1.26, 1.17 (s, 12H, 4CH_3_), 4.63, 4.60, 4.49, 4.31 (m, 4H, 4CH, H_2_, H_3_, H_4_, H_5_), 4.49 (d, 2H, CH_2_–N), 5.26 (d, 2H, CH_2_–N, H_6_), 5.37 (d, 1H, CH, H_1_), 7.49–7.06 (m, 4H, H_arom_), 7.81 (s, 1H, CH_vinyl_), 8.01 (s, 1H, CH_triazole_); ^13^C-NMR (DMSO-_d6_, 62.5 MHz) δ [ppm]: 161.10 (CO), 143.16, 136.53, 134.47, 120.63, 118.22, 109.33, 108.59 (Cq), 134.72 (CH_vinyl_), 130.47 (CH_triazole_), 129.69, 129.12, 127.96, 126.68, 124.75, 124.29, 117.99 (CH_arom_), 95.94, 71.04, 70.56, 70.15, 67.26 (5CH, C_1_, C_2_, C_3_, C_4_, C_5_), 50.64, 41.57 (CH_2_–N), 26.34, 25.98, 25.26, 24.69 (4CH_3_).

#### (2Z)-2-(4-Chlorobenzylidene)-4-[(1′-1″,2″:3″,4″-di-*O*-isopropylidene-α-d-galactopyranosid-6″-yl)-1′,2′,3′-triazol-4′-yl)methyl]-2*H*-1,4-benzothiazin-3-one **9a**

Yield: 61%; brown oil; ^1^H-NMR (DMSO-_d6_, 300 MHz) δ [ppm]: 1.40, 1.31, 1.30, 1.23 (s, 12H, 4CH_3_), 4.69, 4.40, 4.34, 4.24 (m, 4H, 4CH, H_2_, H_3_, H_4_, H_5_), 4.42 (d, 2H, CH_2_–N), 5.55 (d, 2H, CH_2_–N, H_6_), 5.45 (d, 1H, CH, H_1_), 7.65–7.03 (m, 4H, H_arom_), 7.85 (s, 1H, CH_vinyl_), 8.27 (s, 1H, CH_triazole_); ^13^C-NMR (DMSO-_d6_, 62.5 MHz) δ [ppm]: 161.61 (CO), 135.80, 134.83, 134.54, 130.06, 129.51, 119.93, 109.29, 108.61 (Cq), 135.04 (CH_vinyl_), 132.19 (CH_triazole_), 130.31, 129.53, 128.81, 127.85, 126.45, 124.48, 117.83 (CH_arom_), 95.85, 70.91, 70.57, 69.73, 68.12 (5CH, C_1_, C_2_, C_3_, C_4_, C_5_), 48.49, 39.23 (CH_2_–N), 26.29, 25.95, 25.27, 24.72 (4CH_3_).

#### (2Z)-2-(4-Chlorobenzylidene)-4-[(1′-1″,2″:3″,4″-di-*O*-isopropylidene-α-d-galactopyranosid-6″-yl)-1′,2′,3′-triazol-5′-yl)methyl]-2*H*-1,4-benzothiazin-3-one **9b**

Yield: 17%; brown oil; ^1^H-NMR (DMSO-_d6_, 300 MHz) δ [ppm]: 1.39, 1.30, 1.26, 1.18 (s, 12H, 4CH_3_), 4.62, 4.39, 4.28, 4.15 (m, 4H, 4CH, H_2_, H_3_, H_4_, H_5_), 4.55 (d, 2H, CH_2_–N), 5.37 (d, 2H, CH_2_–N, H_6_), 5.30 (d, 1H, CH, H_1_), 7.63–7.04 (m, 4H, H_arom_), 7.82 (s, 1H, CH_vinyl_), 7.99 (s, 1H, CH_triazole_); ^13^C-NMR (DMSO-_d6_, 62.5 MHz) δ [ppm]: 160.82 (CO), 143.06, 136.80, 134.58, 125.30, 120.81, 117.99, 109.90, 108.09 (Cq), 135.03 (CH_vinyl_), 130.06 (CH_triazole_), 129.91, 129.38, 128.50, 126.68, 124.43, 118.22 (CH_arom_), 96.50, 71.42, 70.90, 70.15, 67.62 (5CH, C_1_, C_2_, C_3_, C_4_, C_5_), 50.93, 41.42 (CH_2_–N), 26.05, 26.71, 25.45, 24.98 (4CH_3_).

#### 4-[(1′-2″,3″,4″,6″-Tétra-*O*-acétyl-(d)-glucopyranos-1″-yl)-1′,2′,3′-triazol-4′-yl)methyl]-2*H*-1,4-benzothiazin-3-one **10a**

Yield: 64%; brown oil; ^1^H-NMR (DMSO-_d6_, 300 MHz) δ [ppm]: 2.01, 1.95, 1.92, 1.72 (s, 12H, 4CH_3_), 3.42 (s, 2H, CH_2_–S); 5.68, 5.55, 5.21, 4.08 (m, 5H, 4CH, H_2_, H_3_, H_4_, H_5_), 4.37 (d, 2H, CH_2_–N), 5.32 (d, 2H, CH_2_–O, H_6_), 6.31 (d, 1H, CH, H_1_), 7.61–7.02 (m, 4H, H_arom_), 8.35 (s, 1H, CH_triazole_); ^13^C-NMR (DMSO-_d6_, 62.5 MHz) δ [ppm]: 170.52, 170.24, 169.88, 168.88, 161.52 (5C=O), 144.03, 136.89, 134.50, 120.16 (Cq), 130.50 (CH_triazole_), 127.75, 124.10, 123.41, 118.13 (CH_arom_), 84.64, 73.81, 72.26, 70.70, 68.21 (5CH, C_1_, C_2_, C_3_, C_4_, C_5_), 62.45 (CH_2_–O), 41.84 (CH_2_–N), 30.50 (CH_2_–S), 21.07, 20.82, 20.46, 20.15 (4CH_3_).

#### 4-[(1′-2″,3″,4″,6″-Tétra-*O*-acétyl-(d)-glucopyranos-1″-yl)-1′,2′,3′-triazol-5′-yl)methyl]-2*H*-1,4-benzothiazin-3-one **10b**

Yield: 21%; brown oil; ^1^H-NMR (DMSO-_d6_, 300 MHz) δ [ppm]: 2.01, 1.97, 1.95, 1.72 (s, 12H, 4CH_3_), 3.42 (s, 2H, CH_2_–S); 5.68, 5.55, 5.21, 4.09 (m, 5H, 4CH, H_2_, H_3_, H_4_, H_5_), 4.37 (d, 2H, CH_2_–N), 5.32 (d, 2H, CH_2_–O, H_6_), 6.37 (d, 1H, CH, H_1_), 7.51–7.03 (m, 4H, H_arom_), 7.63 (s, 1H, CH_triazole_); ^13^C-NMR (DMSO-_d6_, 62.5 MHz) δ [ppm]: 170.24, 170.03, 169.75, 168.55, 161.13 (5C=O), 144.23, 136.66, 133.48, 120.78 (Cq), 130.61 (CH_triazole_), 129.29, 128.07, 124.43, 118.13 (CH_arom_), 84.64, 73.81, 72.59, 70.70, 68.21 (5CH, C_1_, C_2_, C_3_, C_4_, C_5_), 62.45 (CH_2_–O), 41.84 (CH_2_–N), 30.51 (CH_2_–S); 20.96, 20.82, 20.68, 20.29 (4CH_3_).

#### (2Z)-2-Benzylidene-4-[(1′-2″,3″,4″,6″-tétra-*O*-acétyl-(d)-glucopyranos-1″-yl)-1′,2′,3′-triazol-4′-yl)methyl]-2*H*-1,4-benzothiazin-3-one 11a

Yield: 66%; brown oil; ^1^H-NMR (DMSO-_d6_, 300 MHz) δ [ppm]: 2.00, 1.97, 1.93, 1.71 (s, 12H, 4CH_3_), 5.65, 5.51, 5.17, 4.07 (m, 5H, 4CH, H_2_, H_3_, H_4_, H_5_), 4.34 (d, 2H, CH_2_–N), 5.30 (d, 2H, CH_2_–O, H_6_), 6.31 (d, 1H, CH, H_1_), 7.84 (s, 1H, CH_vinyl_), 7.62–7.06 (m, 4H, H_arom_), 8.37 (s, 1H, CH_triazole_); ^13^C-NMR (DMSO-_d6_, 62.5 MHz) δ [ppm]: 170.52, 170.04, 169.85, 168.83, 161.13 (5C=O), 144.13, 136.46, 134.53, 120.63, 118.31 (Cq), 130.51 (CH_triazole_), 134.77 (CH_vinyl_), 129.51, 129.09, 127.90, 126.66, 124.27, 123.54, 117.97 (CH_arom_), 84.33, 73.80, 72.58, 70.58, 67.99 (5CH, C_1_, C_2_, C_3_, C_4_, C_5_), 62.28 (CH_2_–O), 41.51 (CH_2_–N), 20.96, 20.82, 20.68, 20.26 (4CH_3_).

#### (2Z)-2-Benzylidene-4-[(1′-2″,3″,4″,6″-tétra-*O*-acetyl-(d)-glucopyranos-1″-yl)-1′,2′,3′-triazol-5′-yl)methyl]-2*H*-1,4-benzothiazin-3-one **11b**

Yield: 20%; brown oil; ^1^H-NMR (DMSO-_d6_, 300 MHz) δ [ppm]: 2.01, 1.97, 1.92, 1.72 (s, 12H, 4CH_3_), 5.64, 5.54, 5.21, 4.09 (m, 5H, 4CH, H_2_, H_3_, H_4_, H_5_), 4.34 (d, 2H, CH_2_–N), 5.30 (d, 2H, CH_2_–O, H_6_), 6.34 (d, 1H, CH, H_1_), 7.84 (s, 1H, CH_vinyl_), 7.65–7.03 (m, 4H, H_arom_), 7.62 (s, 1H, CH_triazole_); ^13^C-NMR (DMSO-_d6_, 62.5 MHz) δ [ppm]: 170.52, 170.24, 169.88, 168.88, 161.39 (5C=O), 144.03, 136.66, 134.56, 120.78, 118.44 (Cq), 130.17 (CH_triazole_), 134.73 (CH_vinyl_), 129.65, 129.29, 127.80, 126.66, 124.43, 123.67, 118.12 (CH_arom_), 84.40, 73.89, 72.59, 70.70, 68.21 (5CH, C_1_, C_2_, C_3_, C_4_, C_5_), 62.45 (CH_2_–O), 41.84 (CH_2_–N), 21.07, 20.82, 20.68, 20.40 (4CH_3_).

#### (2Z)-2-(4-Chlorobenzylidene)-4-[(1′-2″,3″,4″,6″-tetra-*O*-acetyl-(d)-glucopyranos-1″-yl)-1′,2′,3′-triazol-4′-yl)methyl]-2*H*-1,4-benzothiazin-3-one **12a**

Yield: 63%; brown oil; ^1^H-NMR (DMSO-_d6_, 300 MHz) δ [ppm]: 2.01, 1.97, 1.95, 1.72 (s, 12H, 4CH_3_), 5.68, 5.55, 5.14, 4.13 (m, 5H, 4CH, H_2_, H_3_, H_4_, H_5_), 4.37 (d, 2H, CH_2_–N), 5.35 (d, 2H, CH_2_–O, H_6_), 6.34 (d, 1H, CH, H_1_), 7.84 (s, 1H, CH_vinyl_), 7.68–7.06 (m, 4H, H_arom_), 8.39 (s, 1H, CH_triazole_); ^13^C-NMR (DMSO-_d6_, 62.5 MHz) δ [ppm]: 170.52, 170.24, 169.85, 169.22, 161.39 (5C=O), 144.03, 136.66, 134.76, 130.45, 120.78, 118.44 (Cq), 130.51 (CH_triazole_), 134.53 (CH_vinyl_), 129.99, 129.09, 127.80, 126.66, 124.10, 118.12 (CH_arom_), 84.40, 73.1, 72.59, 70.70, 68.21 (5CH, C_1_, C_2_, C_3_, C_4_, C_5_), 62.12 (CH_2_–O), 40.99 (CH_2_–N), 21.07, 20.82, 20.46, 20.06 (4CH_3_).

#### (2Z)-2-(4-Chlorobenzylidene)-4-[(1′-2″,3″,4″,6″-tetra-*O*-acetyl-(d)-glucopyranos-1″-yl)-1′,2′,3′-triazol-5′-yl)methyl]-2*H*-1,4-benzothiazin-3-one **12b**

Yield: 19%; brown oil; ^1^H-NMR (DMSO-_d6_, 300 MHz) δ [ppm]: 2.00, 1,95, 1.92, 1.73 (s, 12H, 4CH_3_), 5.62, 5.48, 5.14, 4.08 (m, 5H, 4CH, H_2_, H_3_, H_4_, H_5_), 4.34 (d, 2H, CH_2_–N), 5.27 (d, 2H, CH_2_–O, H_6_), 6.34 (d, 1H, CH, H_1_), 7.84 (s, 1H, CH_vinyl_), 7.65–7.05 (m, 4H, H_arom_), 7.61 (s, 1H, CH_triazole_); ^13^C-NMR (DMSO-_d6_, 62.5 MHz) δ [ppm]: 170.24, 170.03, 169.46, 168.55, 161.13 (5C=O), 144.55, 136.46, 134.56, 130.57, 120.16, 118.57 (Cq), 130.50 (CH_triazole_), 134.17 (CH_vinyl_), 129.47, 129.09, 127.80, 126.66, 124.10, 118.12 (CH_arom_), 84.06, 73.23, 72.54, 70.24, 68.00 (5CH, C_1_, C_2_, C_3_, C_4_, C_5_), 62.12 (CH_2_–O), 41.74 (CH_2_–N), 20.96, 20.82, 20.74, 20.29 (4CH_3_).

#### 4-[1,2,3-Triazolylmethyl]-2*H*-1,4-benzothiazin-3-one **13**

Yield: 79%; mp = 352 K; ^1^H-NMR (DMSO-_d6_, 300 MHz) δ [ppm]: 7.40 (s, 1H, CH_triazole_), 7.37–7.00 (m, 4H, H_arom_), 5.16 (d, 2H, CH_2_–N), 3.56 (s, 2H, CH_2_–S); ^13^C-NMR (DMSO-_d6_, 62.5 MHz); 165.49 (CO), 143.56, 139.75, 123.44 (Cq), 128.50 (CH_triazole_), 129.11, 127.72, 123.93, 118.65 (CH_arom_), 40.32 (C–N), 30.76 (C–S).

#### (2Z)-2-Benzylidene-4-[1,2,3-triazolylmethyl]-2*H*-1,4-benzothiazin-3-one **14**

Yield: 81%; brown oil; ^1^H-NMR (DMSO-_d6_, 300 MHz) δ [ppm]: ^1^H-NMR (DMSO-_d6_, 300 MHz) δ [ppm]: 7.84 (s, 1H, CH_vinyl_), 7.70–7.10 (m, 9H, H_arom_), 7.54 (s, 1H, CH_triazole_), 4.86 (d, 2H, CH_2_–N); ^13^C-NMR (DMSO-_d6_, 62.5 MHz); 160.79 (CO), 136.01, 134.48, 133.51, 121.18, 118.42 (Cq), 134.28 (CH_vinyl_), 128.40 (CH_triazole_), 134.28, 132.55, 129.12, 128.40, 126.95, 124.73, 118.05 (CH_arom_), 35.47 (C–N).

#### (2Z)-2-(4-Chlorobenzylidene)-4-[1,2,3-triazolyl-methyl]-2*H*-1,4-benzothiazin-3-one **15**

Yield: 77%; brown oil; ^1^H-NMR (DMSO-_d6_, 300 MHz) δ [ppm]: 7.83 (s, 1H, CH_vinyl_), 7.67–7.10 (m, 8H, H_arom_), 7.53 (s, 1H, CH_triazole_), 4.85 (d, 2H, CH_2_–N); ^13^C-NMR (DMSO-_d6_, 62.5 MHz); 160.68 (CO), 135.77, 134.28, 133.31, 132.29, 121.05, 118.05 (Cq), 134.15 (CH_vinyl_), 128.14 (CH_triazole_) 132.30, 129.12, 128.17, 126.86, 124.73, 117.85 (CH_arom_), 35.01 (C-N).

#### Ethyl-*n*-(benzoyl)-2-ethoxylglycinate **16**

Yield: 78%; mp = 369. ^1^H-NMR (DMSO-_d6_, 300 MHz) δ [ppm]: 9.42 (d, 1H, N–H, J = 9,41), 7.92–7.44 (m, 5H, H arom), 5.62 (d, 1H, CH, J = 5,61), 4.13 (q, 2H, CH2–O), 3.57 (q, 2H, CH 2–O), 1.19, 1.13 (t, 6H, 2CH3); 13 C-NMR (DMSO-d6, 62.5 MHz); 168.47, 167.12 (2 CO), 133.54, 132.48, 128.87, 128.27 (CH_arom_), 77.94 (CH), 63.70, 61.62 (2CH_2_), 15.38, 14.46 (2CH_3_).

## References

[1] Trapani G, Reho A, Morlacchi F, Latrofa A, Marchini P, Venturi F, Cantalamessa F (1985). Synthesis and antiinflammatory activity of various 1,4-benzothiazine derivatives. Farmaco Ed Sci.

[2] Gowda J, Khader AMA, Kalluraya B, Shree P, Shabaraya AR (2011). Synthesis, characterization and pharmacological activity of 4-{[1-substituted aminomethyl-4-arylideneamino-5-sulfanyl-4,5-dihydro-1H-1,2,4-triazol-3-yl] methyl}-2*H*-1,4-benzothiazin-3(4*H*)-ones. Eur J Med Chem.

[3] Warren BK, Knaus EE (1987). Pyridine and reduced pyridine analogues of 10*H*-pyrido [3,4-b][1, 3] benzothiazines with analgesic activity. Eur J Med Chem.

[4] Armenise D, Muraglia M, Florio MA, Laurentis ND, Rosato A, Carrieri A, Corbo F, Franchini C (2012). 4*H*-1,4-Benzothiazine, dihydro-1,4-benzothiazinones and 2-amino-5-fluorobenzenethiol derivatives: ciprofloxacin-treated mammalian cells. Mol Pharmacol.

[5] Rathore BS, Kumar M (2006). Synthesis of 7-chloro-5-trifluoromethyl/7-fluoro/7-trifluoromethyl-4*H*-1,4-benzothiazines as antimicrobial agents. Bioorg Med Chem.

[6] Sabatini S, Kaatz GW, Rossolini GM, Brandim D, Fravolini A, Rossolini GM, Brandini D, Fravolini A (2008). From phenothiazine to 3-phenyl-1,4-benzothiazine derivatives as inhibitors of the *Staphylococcus aureus* NorA multidrug efflux pump. J Med Chem.

[7] Fringuelli R, Schiaffella F, Bistoni F, Pitzurra L, Vecchiarelli A (1998). Azole derivatives of 1,4-benzothiazine as antifungal agents. Bioorg Med Chem.

[8] Malagu K, Boustie J, David M, Sauleau J, Amoros M, Girre RL, Sauleau A (1998). Synthesis and antiviral activity of new 1,4-benzothiazines: sulphoxides and sulphone derivatives. Pharm Pharmacol Commun.

[9] Takemoto I, Yamasaki K, Kaminaka H (1994). Synthesis of a fluorobenzoxazine derivative and its analogues. Biosci Biotechnol Biochem.

[10] Gupta RR, Kumar R (1986). Synthesis of 6-trifluoromethyl-4*H*-1,4-benzothiazines as possible anticancer agents. J Fluorine Chem.

[11] Gupta V, Gupta RR (1991). Single step synthesis of fluorinated 4-*H*-1,4-benzothiazines as possible anticancer agents. J Prakt Chem.

[12] Gupta RR, Kumar R, Gautam RK (1985). Synthesis of new fluorinated 4*H*-1,4-benzothiazines as possible anticancer agents. J Fluorine Chem.

[13] Jacquot Y, Bermont L, Giorgi H, Refouvelet B, Adessi GL, Daubrosse E, Xicluna A (2001). Substituted benzopyranobenzothiazinones. Synthesis and estrogenic activity on MCF-7 breast carcinoma cells. Eur J Med Chem.

[14] Zia-ur-Rehman M, Choudary JA, Elsegood MRJ, Siddiqui HL, Khan KM (2009). A facile synthesis of novel biologically active 4-hydroxy-*N*′-(benzylidene)-2*H*-benzo[e][1, 2]thiazine-3-carbohydrazide 1,1-dioxides. Eur J Med Chem.

[15] Vidal A, Madelmont JC, Mounetou EA (2006). Simple and efficient synthesis of methyl 3,4-dihydro-2-methyl-2*H*-1,2-benzothiazine-3-carboxylate 1,1-dioxide from saccharin sodium salt. Synthesis.

[16] Tawada H, Sugiyama Y, Ikeda H, Yamamoto Y, Meguro K (1990). Studies on antidiabetic agents. IX. A new aldose reductase inhibitor, AD-5467, and related 1,4-benzoxazine and 1,4-benzothiazine derivatives: synthesis and biological activity. Chem Pharm Bull.

[17] Ellouz M, Sebbar NK, Elmsellem H, Steli H, Fichtali I, Mohamed AMM, Mamari KA, Essassi EM, Abdel-Rahaman I (2016). Inhibitive properties and quantum chemical studies of 1,4-benzothiazine derivatives on mild steel corrosion in acidic medium. J Mater Environ Sci.

[18] Ellouz M, Elmsellem H, Sebbar NK, Steli H, Al Mamari K, Nadeem A, Ouzidan Y, Essassi EM, Abdel-Rahaman I, Hristov P (2016). Anti-corrosive properties of benzothiazine derivatives on mild steel corrosion in 1M HCl solution: experimental and theoretical studies. J Mater Environ Sci.

[19] McDouall JJW, Robb MA, Niazi U, Bernardi F, Schlegel HB (1987). An MCSCF study of the mechanisms for 1,3 dipolar cycloadditions. J Am Chem Soc.

[20] Ahabchane NH, Essassi EM, Lopez L, Bellan J, Lamandé LCR (2000). Synthese de 2-pyrazolinyl, isoxazolinyl, 1,2,3-triazolyl et 1,3,4-oxadiazolyl methylmercapto-1-pyrazolyl benzimidazole. Acad Sci Paris Série IIc.

[21] Azzaoui BE, Rachid B, Doumbia ML, Essassi EM, Gornitzka H, Bellan J (2006). Unexpected opening of benzimidazole derivatives during 1,3-dipolar cycloaddition. Tetrahedron Lett.

[22] Sebbar NK, Zerzouf A, Essassi EM, Saadi M, Ammari L (2014). 4-(Prop-2-ynyl)-2H-1,4-benzothiazin-3(4*H*)-one. Acta Cryst E.

[23] Sebbar NK, Zerzouf A, Essassi EM, Saadi M, Ammari LE (2014). (2Z)-2-Benzylidene-4-(prop-2-yn-1-yl)-2*H*-1,4-benzothiazin-3(4*H*)-one. Acta Cryst E.

[24] Da Silva FDC, De Souza MCBV, Frugulhetti IIP, Castro HC, Souza SLDO, De Souza TML, Rodrigues DQ, Souza AMT, Abreu PA, Passamani F, Rodrigues CR, Ferreira VF (2009). Synthesis, HIV-RT inhibitory activity and SAR of 1-benzyl-1*H*-1,2,3-triazole derivatives of carbohydrates. Eur J Med Chem.

[25] Giffin MJ, Heaslet H, Brik A, Lin YC, Cauvi G, Wong C-H, McRee DE, Elder JH, Stout CD, Torbett BE (2008). A copper(I)-catalyzed 1,2,3-triazole azide alkyne click compound is a potent inhibitor of a multidrug-resistant HIV-1 protease variant. J Med Chem.

[26] Gulevich AV, Dudnik AS, Chernyak N, Gevorgyan V (2013). Transition metal-mediated synthesis of monocyclic aromatic heterocycles. Chem Rev.

[27] Dardouri R, Rodi YK, Haoudi A, Mazzah A, Skalli MK, Essassi EM, Ouazzani CF (2012). Synthèse et modélisation de nouveaux systèmes hétérocycliques obtenus par cycloaddition 1,3-dipolaire dérivant de la 1,5-benzodiazépine-2,4-dione. J Mar Chim Heterocycl.

[28] Ahabchane NH, Keita A, Essassi EM (1999) Synthèse des 1-pyrazolyl, isoxazolyl et 1,2,3-triazolylméthyl-1,5-benzodiazépine par cycloaddition dipolaire-1,3. C R Acad Sci Paris Série IIc:591

[29] Alaoui IC, Rodi YK, Keita A, Sabir S, Skalli MK, El Hadrami EM, Essassi EM (2008). Synthesis of new heterocyclic systems by 1,3-dipolar cycloaddition from the 1,5-benzodiazepine-2,4-dione. Phys Chem News.

[30] Sebbar NK, Mekhzoum MEM, Essassi EM, Zerzouf A, Talbaoui A, Bakri Y, Saadi M, Ammari LE (2016). Novel 1,4-benzothiazine derivatives: synthesis, crystal structure, and anti-bacterial properties. Res Chem Intermed.

[31] Wang Q, Chan TR, Hilgraf R, Fokin VV, Scharpless KB, Finn M (2003). Bioconjugation by copper(I)-catalyzed azide-alkyne [3 + 2] cycloaddition. J Am Chem Soc.

[32] Bock VD, Hiemstra H, Van Maarseveen JH (2006). CuI-catalyzed alkyne–azide click cycloadditions from a mechanistic and synthetic perspective. Eur J Org Chem.

[33] Tornøe CW, Christensen C, Meldal M (2002). Peptidotriazoles on solid phase: [1,2,3]-triazoles by regiospecific copper(I)-catalyzed 1,3-dipolar cycloadditions of terminal alkynes to azides. J Org Chem.

[34] Himo F, Lovell T, Hilgraf R, Rostovtsev VV, Noodleman L, Sharpless KB, Fokin VV (2005). Copper (I)-catalyzed synthesis of azoles. DFT study predicts unprecedented reactivity and intermediates. J Am Chem Soc.

[35] Zaidan MRS, Noor Rain A, Badrul AR, Adlin A, Norazah A, Zakiah I (2005). In vitro screening of five local medicinal plants for antibacterial activity using disc diffusion method. Trop Biomed.

[36] Brik A, Alexandratos J, Lin YC, Elder JH, Olson AJ, Wlodawer A, Goodsell DS, Wong C-H (2005). 1,2,3-Triazole as a peptide surrogate in the rapid synthesis of HIV-1 protease inhibitors. ChemBioChem.

[37] Fichtali I, Chraibi M, Aroussi FE, Ben-Tama A, Hadrami EME, Benbrahim KF, Stiriba SE (2016). Synthesis of some 1,2,3-triazoles derivatives and evaluation of their antimicrobial activity. Der Pharma Chem.

[38] Abdel-Wahab BF, Mohamed HA, Awad GEA (2015). Synthesis and biological activity of some new 1,2,3-triazole hydrazone derivatives. Eur Chem Bull.

